# Calcineurin A *versus* NS5A-TP2/HD Domain Containing 2: A Case Study of Site-directed Low-frequency Random Mutagenesis for Dissecting Target Specificity of Peptide Aptamers*[Fn FN2]

**DOI:** 10.1074/mcp.M112.024612

**Published:** 2013-04-10

**Authors:** Silvia Dibenedetto, David Cluet, Pierre-Nicolas Stebe, Véronique Baumle, Jérémie Léault, Raphaël Terreux, Marc Bickle, Benoit D. E. Chassey, Ivan Mikaelian, Pierre Colas, Martin Spichty, Michele Zoli, Brian B. Rudkin

**Affiliations:** From the ‡Differentiation & Cell Cycle Group, Laboratoire de Biologie Moléculaire de la Cellule, UMR5239, Centre National pour la Recherche Scientifique (CNRS), Ecole Normale Supérieure de Lyon, Université Lyon 1, SFR BioSciences Gerland-Lyon Sud UMS3444-US8, 46 Allée d'Italie, 69007 Lyon, France;; §Department of Biomedical, Metabolic and Neural Sciences, University of Modena and Reggio Emilia, via Campi 287, 41125 Modena, Italy;; ‖Institut de Biologie et Chimie des Protéines – UMR5086 – CNRS, Université Lyon 1, 7, Passage du Vercors, 69 367 Lyon, Cedex 07, France;; **Aptanomics, S.A., 181-203 avenue Jean Jaures, 69007 Lyon, France

## Abstract

We previously identified a peptide aptamer (named R5G42) via functional selection for its capacity to slow cell proliferation. A yeast two-hybrid screen of human cDNA libraries, using R5G42 as “bait,” allowed the identification of two binding proteins with very different functions: calcineurin A (CnA) (PP2B/PPP3CA), a protein phosphatase well characterized for its role in the immune response, and NS5A-TP2/HD domain containing 2, a much less studied protein induced subsequent to hepatitis C virus non-structural protein 5A expression in HepG2 hepatocellular carcinoma cells, with no known activity. Our objective in the present study was to dissect the dual target specificity of R5G42 in order to have tools with which to better characterize the actions of the peptide aptamers toward their individual targets. This was achieved through the selection of random mutants of the variable loop, derived from R5G42, evaluating their specificity toward CnA and NS5A-TP2 and analyzing their sequence. An interdisciplinary approach involving biomolecular computer simulations with integration of the sequence data and yeast two-hybrid binding phenotypes of these mutants yielded two structurally distinct conformers affording the potential molecular basis of the binding diversity of R5G42. Evaluation of the biological impact of CnA- *versus* NS5A-TP2-specific peptide aptamers indicated that although both contributed to the anti-proliferative effect of R5G42, CnA-binding was essential to stimulate the nuclear translocation of nuclear factor of activated T cells, indicative of the activation of endogenous CnA. By dissecting the target specificity of R5G42, we have generated novel tools with which to study each target individually. Apta-C8 is capable of directly activating CnA independent of binding to NS5A-TP2 and will be an important tool in studying the role of CnA activation in the regulation of different signaling pathways, whereas Apta-E1 will allow dissection of the function of NS5A-TP2, serving as an example of the usefulness of peptide aptamer technology for investigating signaling pathways.

Our understanding of biological processes is becoming more and more dependent on the global, unbiased evaluation of signaling networks and their regulation. This is achieved through diverse approaches, such as the analysis of global gene transcription and miRNA expression; the evaluation of protein expression; the mapping of protein interactions with proteins, nucleic acids, and lipids; and the flux of metabolites through these pathways. Such research has given rise to the fields of transcriptomics, proteomics, lipidomics, and metabolomics. As the complexity increases, so does the need for focused and unbiased means of identifying key players in the pathways. This identification can be achieved to some extent, for example, through analysis of the impact of disease-related mutations (hereditary or environmental), as well as through the use of siRNA screens (1) or other random (2, 3) or targeted knock-out approaches. However, although these analyses can offer an idea of which genes or proteins might be important, if we wish to enhance our understanding of the underlying mechanisms it is necessary to move to the next level of complexity: knowledge of the protein function and, subsequently, which partners of the proteins are involved. The implementation of peptide aptamer technology offers a means of addressing these aspects.

Peptide aptamers are combinatorial proteins that consist of a variable loop constrained within a constant scaffold protein (4). They are conceived to selectively interact with specific targets and modulate their function. The high specificity of their binding to the target enables discrimination between closely related members of a protein family (4) or proteins that differ by a single point mutation (5, 6). Thanks to their ability to modulate the function of a given protein or protein–protein interaction, they are used as precision tools for the dissection of signaling pathways (7, 8). Moreover, peptide aptamer–target interactions can be used to identify small molecules that bind to the target and displace the peptide aptamer (5, 9). These, in turn, can become precursors for the development of leads for research purposes or for pre-clinical development (10).

We have previously identified, through functional selection, a peptide aptamer, R5G42, that slows cell proliferation in culture (11). Two proteins have been isolated as targets of this peptide aptamer: calcineurin A (CnA)^1^ and hepatitis C virus NS5A-transactivated protein 2 (NS5A-TP2) (12). The present study aimed at investigating the contribution of both interactions to cell proliferation. This involved the development and characterization of peptide aptamers derived from R5G42 that are exclusively specific for one or the other of these targets.

NS5A-TP2 is also known as HD domain containing 2 (HDDC2). The HD domain named HD—after the conserved doublet of predicted catalytic residues, defines a super-family of proteins with cation-dependent phosphodiesterase activity (13). This family includes proteins with different HD domain architecture, phylogenetic distributions, and, above all, biological functions. Despite the heterogeneity of this family, the proteins seem to be involved in the same types of processes, such as nucleic acid metabolism and signal transduction (13). Regarding NS5A-TP2, analysis of the transcriptome in various organisms, including humans, has revealed that the expression of this protein is modulated under certain pathophysiological conditions—for example, it is down-regulated in pterygia, an affliction of the cornea (14); it is a candidate gene for resistance to infectious salmon anemia, the “hoof and mouth” disease equivalent for salmon fisheries (15); and it belongs to a cluster of SNPs responsible for differences between individuals' dependence on alcohol (16)—but so far, little is known about the specific biological role of this protein.

Calcineurin is a well-characterized protein serine/threonine phosphatase that plays a key role in several cellular responses (17, 18). The best-described role of calcineurin is its critical participation in immune response activation via nuclear translocation of the transcription factor, nuclear factor of activated T cells (NFAT). Furthermore, calcineurin and NFAT have been shown to participate in signaling cascades that govern the development and function of the nervous system (19), skeletal muscle tissue (20), and cardiovascular system (21).

Calcineurin is a heterodimer composed of a catalytic subunit of 61 kDa, CnA, and a regulatory subunit of 19 kDa, calcineurin B (17). The catalytic subunit contains several distinct functional regions: a catalytic domain (residues 71–342), a calcineurin B binding domain (residues 346–370), a calmodulin (CaM) binding domain (residues 391–414), and an auto-inhibitory (AI) domain (residues 457–482). In the inactive form of the enzyme, the AI domain blocks the active site, preventing access to the substrate. An increase in the intracellular calcium concentration leads to the activation of CaM and, through subsequent interaction with the CaM binding domain of CnA, triggers a conformational change that displaces the AI domain. The catalytic site of the enzyme thus becomes available for the substrate (22).

Because of the importance of CnA in the transduction of Ca^2+^ signaling in a cell, modulation of its activity has been the focus of numerous studies over the years, with a view to developing therapeutic approaches for the treatment of several human pathologies. CnA has been shown to be a target of two widely used immunosuppressive drugs, cyclosporin A and FK506 (Tacrolimus) (23, 24). These drugs are used to inhibit the phosphatase activity of CnA and the subsequent activation of the immune response in order to prevent the rejection of transplanted organs.

Although molecules able to *inhibit* calcineurin activity have been well characterized, to our knowledge, peptide aptamer R5G42 is the first and only exogenous molecule reported to be capable of the direct binding and *activation* of endogenous calcineurin (see, however, Ref. 25).

Several studies propose that the activation of calcineurin could be beneficial in the treatment of skeletal muscle disease (26) and neurodegenerative disease (27, 28). These conclusions were obtained, however, in an indirect manner, either through the forced expression of constitutively activated CnA or observation of the effect of the inhibitors described above. The peptide aptamer R5G42 can be considered as a guide for the development of a molecule that could be used for investigating the therapeutic potential of direct activation of endogenous CnA. However, its dual target specificity represents a major challenge that must be resolved first. Thus, the identification of peptide aptamers specific for one or the other target protein would allow a more precise dissection of the cellular response.

In order to optimize the binding to CnA and abolish that to NS5A-TP2 (and vice versa), a library of peptide aptamers derived from R5G42 has been generated via low frequency region-specific random mutagenesis (29) of the variable loop and the selection of mutants exclusive for one or the other target binding. An integrated approach involving biomolecular computer simulations coupled with the sequence data and yeast two-hybrid phenotypes has been applied in order to identify potential structural determinants of the variable loop that could distinguish between the two targets. Moreover, we have identified the minimal binding domain on CnA, of a CnA exclusive peptide aptamer, and we further discuss a possible model for its action on the activation of calcineurin.

## EXPERIMENTAL PROCEDURES

### 

#### 

##### Plasmids

For the yeast two-hybrid (Y2H) assay, the peptide aptamers were cloned as bait in the plasmid pGilda, in fusion with the *E. coli* LexA repressor and under control of the galactose-inducible Gal4 promoter, whereas CnA (385–520) and NS5A-TP2 were used as prey in the pJG4–5 plasmid (30) (Clontech; Genbank Accession Number U89961) as described elsewhere (11). The prey vector pWP2, coding for RG22, an anti-LexA peptide aptamer, and the pWP2-C5 vector coding for a non-relevant peptide aptamer were used, respectively, as positive and negative controls. The pSH18–34 plasmid was used as a reporter vector, bearing a beta-galactosidase gene under the control of four overlapping LexA operators (31).

For the co-immunoprecipitation, peptide aptamers were cloned into the mammalian pCI-HA plasmid (derived from Promega pCI) under the control of a CMV promoter. Full-length CnA and NS5ATP2 were expressed using another derivative vector of pCI, pX2-M2, having the same expression cassette as pCI-HA but with the M2 tag instead of HA, and allowing cloning by homologous recombination in yeast via the insertion of the HIS3 gene and the CEN sequence of pEG202 in the ClaI restriction site of the pCI plasmid.

Evaluation of the localization of the transcription factor NFATc1 was achieved using a plasmid containing a full-length wild-type NFATc1 linked to E-GFP (NFATc1-GFP) (32). Peptide aptamers and a truncated (1–390) constitutively active form of calcineurin (CnA*) were cloned into the pCI derivative plasmid pM2, allowing co-expression of a dual HA-and-M2 tagged protein at its N-terminal part with mCherry under the control of the encephalomyocarditis virus internal ribosome entry site isolated from the pVRV6 plasmid (11).

##### Random Mutagenesis

Specific random mutagenesis on the variable loop of the peptide aptamer R5G42 was performed using the following primers: forward: 5′-AAACTTGTAGTAGTTGACTTCTCAGCCACG-3′; and reverse: 5′-AGAGAGGGAATGAAAGAAAGGCTTGATCAT-3′. In order to generate low frequency mutations, PCR was performed with 0.25 mm of dATP and dGTP and with 1.25 mm of dCTP and dTTP, using taq polymerase (New England BioLabs, Ipswich, MA) (29). The PCR products were then cloned using homologous recombination in yeast (33, 34); pEG202-hTrx opened in the active site of hTrx using RsrII digestion. A mutant library was then created by picking isolated clones that were subsequently sequenced and characterized for CnA (385–520) and/or NS5A-TP2 binding. The Y2H assay was performed using TB50-alpha and EYG42 yeast strains as described elsewhere (35) to verify the phenotype of the interaction with each target.

##### Cell Culture

HeLa cells (ATCC number: CCL-2) and XC cells (ATCC number: CCL-165) were maintained in Dulbecco's modified Eagle's medium (Invitrogen, France) supplemented with 10% (v/v) heat-inactivated fetal calf serum (Sigma, France) and 1% (v/v) penicillin/streptomycin solution (Invitrogen) at 37 °C in 5% CO_2_. For immunofluorescence, cells were cultured on glass coverslips.

##### Immunoprecipitation

The day prior to transfection, two 100-mm Petri dishes were seeded with 3.125 million HeLa cells per target/peptide aptamer couple. The following day, cells were transfected using 22 μg of peptide aptamer coding vector (pCI-HA) and 9 μg of target coding plasmid (pX2-M2) mixed with 62 μg Lipofectamine 2000 (Invitrogen, Ref. 11668–019). After 6 h of incubation, the culture medium was replaced by a fresh one. Cells were collected 24 h after the beginning of the transfection procedure. Briefly, the culture media of the two dishes for one condition were harvested and pooled. Cells were then treated for 5 min with 2 ml of PBS 10 mm EDTA per dish and harvested and pooled with the culture medium. After three washes with 5 ml of PBS (with calcium and magnesium), cell pellets were resuspended in 1 ml lysis buffer (50 mm Tris HCl, pH 7.4, with 150 mm NaCl, 1 mm EDTA, and 1% Triton X-100) with protease inhibitors (complete EDTA-free protease inhibitor mixture, Roche). After three freezing/thawing cycles, cell suspensions were subjected to three cycles of 30 s of sonication using a Bioruptor at maximum output. Proteins and debris were separated after 10 min centrifugation at 19,000 × *g* in a fixed-angle rotor (Jouan benchtop centrifuge MR1822). Supernatants were then collected and protein concentrations evaluated using the Bradford technique (Bio-Rad).

Co-immunoprecipitation assays were performed after 30 min on ice with 1 mg of total protein in 1 ml of lysis buffer with complete protease inhibitor mixture and 50 μl anti-HA Myltenyi magnetic beads (Ref. 130–091-122). Immunoprecipitates were then washed and collected using Myltenyi MS columns following the supplier's recommendations. Samples were separated on a discontinuous gradient (12%/15%) SDS gel with 29:1 acrylamide/bis-acrylamide and transferred onto Optitran BA-S 83 reinforced nitrocellulose membranes (Whatman, Schleicher & Schule, Maidstone, UK) via total emersion electrotransfer (Bio-Rad). Membranes were subsequently blocked overnight at 4 °C with microfiltered Tris-buffered saline (25 mm Tris, 150 mm NaCl) with 0.1% Tween 20 (TBST) and 5% bovine serum albumin (Sigma, A3294), pH 7.4. Peptide aptamers and targets were revealed using anti-HA and anti-M2 mouse antibodies (Sigma, Refs. H3663 and F3165, 1/1000) diluted in TBST with bovine serum albumin as described above via 1 h of incubation at room temperature followed by three washes with TBST. Membranes were subsequently incubated with goat anti-mouse IgG DyLight 800 (Fisher, Ref. 35521, 1/15,000), following the same procedure as for the primary antibodies. Membranes were then observed using a Li-Cor Odyssey imaging system.

##### Confocal Microscopy

48 h after transfection with appropriate plasmids, cells were rinsed with ice-cold PBS, fixed in 4% paraformaldehyde for 10 min at 20 °C, and permeabilized with 0.3% saponin for 30 min at 20 °C. Cells were labeled with HA-specific primary antibody (see above) and then with donkey anti-mouse Alexa Fluor 555 (Invitrogen, Ref. A31570) in order to visualize the peptide aptamers Trx and CnA*. Coverslips were rinsed in PBS and then dipped in Millipore water before mounting with Vectashield containing DAPI (Vector Laboratories, Burlingame, CA, Ref. H-1200). Slides were observed using a TCS SP5 AOBS DM6000 spectral confocal microscope (Leica, Wetzlar, Germany) with a 40× objective. DAPI was excited with a 405 nm diode laser, GFP with an argon 458/488/514 nm laser, and Alexa Fluor 555 with a helium/neon 633 nm laser. Image acquisition was achieved using the manufacturer's software (Leica LAS AF SP5).

Evaluation of the NFAT-GFP content in each nucleus (RGB signal, 8-bit 0–255) was achieved using the ImageJ program. Mean fluorescence values of NFAT-GFP present in nuclei were obtained for each peptide aptamer and CNA*. The results presented are the mean values calculated for representative cells (*n* = 16 to *n* = 34).

##### Proliferation Assay

XC cells were washed with PBS and diluted in complete culture medium to 5 × 10^5^ cells/ml and then incubated with 10 μm Cell Tracker™ Orange 5-(and-6)-(((4-chloromethyl)benzoyl)amino)tetramethylrhodamine (CMTMR) (Invitrogen, Ref. C2927) for 30 min at 37 °C. They were then incubated in complete culture medium for another 30 min at 37 °C. Cells were then plated onto six-well plates (4 × 10^5^ cells in each well). 4 × 10^5^ cells were washed with PBS, fixed with PBS-1,5% paraformaldehyde, and stored at 4 °C as a reference for the starting point of the proliferation assay. The next day, the transfection procedure was performed with 10 μl of Lipofectamine 2000 and 5 μg of plasmid DNA (pCI-HA series) per well (two wells per condition). When the transfection reaction was started, three wells of non-transfected cells were collected and processed as the reference control. They were used to evaluate CMTMR labeling reproducibility and as proliferation controls (*t* = 0 h). After one night of transfection, the medium was replaced with a fresh one, and 4 h later one well of each transfection was processed and stored (*t* = 24 h). The next day, the contents of the last wells were collected, processed, and stored (*t* = 48 h). All samples were then permeabilized with 0.3% saponin for 30 min at 20 °C. Cells were then labeled with HA-specific primary antibody and goat anti-mouse Cy5 (Abcam, Cambridge, UK, Ref. ab97037) to allow monitoring of peptide aptamer expression. CMTMR and Cy5 fluorescence were subsequently acquired using a FacsCalibur four-color flow cytometer (Beckman Coulter) and analyzed using Cell Quest software (Beckman Coulter).

Analyses were performed on living cells at the time of transfection (*t* = 0 h) and living cells expressing the various peptide aptamers or Trx at *t* = 48 h post-transfection. Anti-proliferative activity was determined by measurement of the geometric mean of the CMTMR labeling in transfected cells (revealed by peptide aptamer or Trx expression). For each condition, cell proliferation was calculated using the following formula:

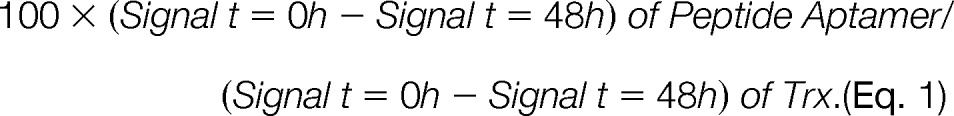


Thus, the maximum proliferation score (100) was attributed to Trx expressing cells. A zero proliferation score (0) was attributed to the CMTMR signal before transfection. The proliferation of cells transfected with R5G42, Apta-C8, and Apta-E1 was then calibrated using these values. Four independent experiments were performed; one major outlier was removed for the analysis of Apta-C8. The statistical significance of the effect of each peptide aptamer *versus* the proliferation observed in cells transfected with Trx was evaluated with a *t* test (see the section “Statistical Analyses”).

##### Computer Simulations (General)

Biomolecular computer simulations were carried out with the program CHARMM (36), version c36b1. The peptide aptamer R5G42 and its mutations S35A, S35P, F39I, and F39Y were modeled in their reduced forms with CHARMM's polar-hydrogen topology, applying the implicit solvation model EEF1 (37). The coordinates of the thioredoxin scaffold are based on the protein database entry 4TRX (38); the unknown coordinates of the loop were constructed initially with the command “IC BUILD” and subsequently with 5000 steps of adopted basis Newton-Raphson minimization (keeping the scaffold fixed). Dihedral potentials were applied during the minimization to avoid cis/trans isomerization in the backbone. All residues of the scaffold that had no atom within 15 Å of the disulfide bond were kept fixed for the following conformational sampling; all other residues were allowed to move freely. Standard non-bonded parameters for EEF1 solvation were used. For the molecular dynamics simulations, the hydrogen masses were set at 4 amu, and all bonds were constrained to their minimum energy value (39). This setup allowed an integration time step of 4 fs. The temperature was controlled via Langevin dynamics with a friction coefficient of 4 ps^−1^.

##### Parallel Tempering

A molecular-dynamics-based parallel tempering algorithm was used to sample the canonical ensemble of R5G42 and its mutations (40). Parallel tempering was performed with 24 heat baths ranging from 290 K to 520 K (*T_n_* = 290 + 10*n*, with *n* = 0, 1, 2, …, 23). The total length of the molecular-dynamics-based parallel tempering simulation was 1.2 μs. Every 20 ps we attempted to swap either all the baths (*T*_2*i*_; *T*_2*i*+1_) or all the baths (*T*_2*i*+1_; *T*_2*i*_), with *i* being an integer; “or” indicates that the selection was made randomly. In total there were 60,000 swap attempts. We saved snapshots every 20 ps for the last 1 μs and obtained ensembles of 50,000 snapshots (200 ns served as equilibration).

##### Clustering

We combined the structures of the heat bath at 310 K from all peptide aptamers, and similar conformations were grouped into clusters by the program Wordom (41), using as a distinction criterion the *C*^α^,*C*^β^-root-mean-square deviation with a cutoff value of 2 Å (with superposition of the atoms that were fixed during the sampling simulations). The data were split into four blocks, each representing 250 ns of parallel tempering, and errors in the population of the clusters (herein called loop conformers) were calculated from the standard error of the mean. Loop conformers with a small population of low statistical significance (twice the standard error of the mean population) were regarded as not populated. For the populated loop conformers, we calculated the free-energy difference relative to the most populated loop conformer of each peptide aptamer, −*kT*ln(*P*_i_/*P*_max_), with *k* being the Boltzmann constant, *T* the temperature (310 K), and *P*_i_ and *P*_max_ the population of the conformer of interest and the most populated conformer, respectively.

##### Statistical Analysis

For the results presented in Table I and Figs. 9 and 10, the mean and standard error of the mean (SEM) were determined. The SEM was calculated using the formula

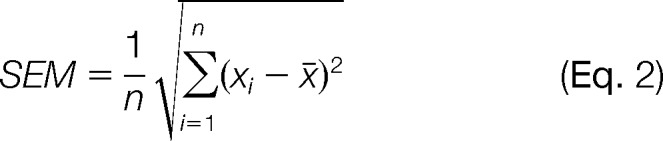

in which *x_i_* is the value of the *i*th observation, *x̄* is the sample mean, and *n* is the sample size.

**Table I:**
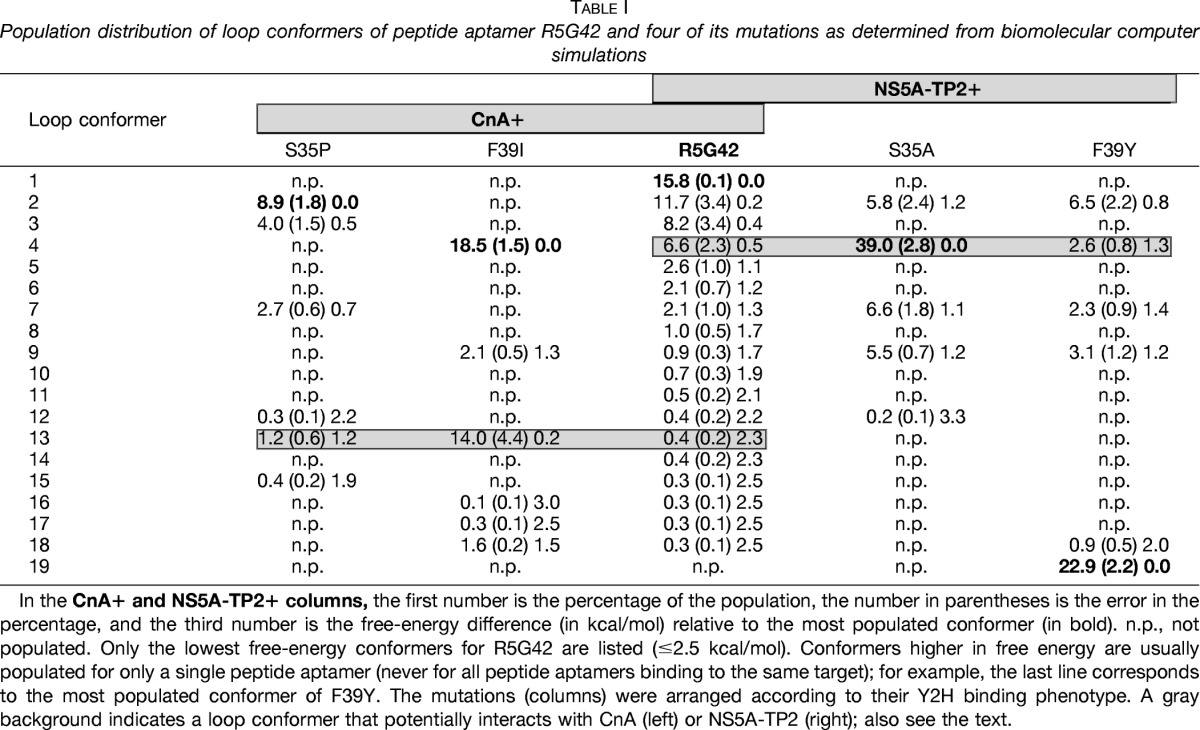
Population distribution of loop conformers of peptide aptamer R5G42 and four of its mutations as determined from biomolecular computer simulations

In the **CnA+ and NS5A-TP2+ columns,** the first number is the percentage of the population, the number in parentheses is the error in the percentage, and the third number is the free-energy difference (in kcal/mol) relative to the most populated conformer (in bold). n.p., not populated. Only the lowest free-energy conformers for R5G42 are listed (≤2.5 kcal/mol). Conformers higher in free energy are usually populated for only a single peptide aptamer (never for all peptide aptamers binding to the same target); for example, the last line corresponds to the most populated conformer of F39Y. The mutations (columns) were arranged according to their Y2H binding phenotype. A gray background indicates a loop conformer that potentially interacts with CnA (left) or NS5A-TP2 (right); also see the text.

For the data presented in Fig. 10, we used *t*-statistics to test the hypothesis that the observed mean value of cell proliferation for a given peptide aptamer *j* is smaller than the reference value of thioredoxin (normalized to 100). The *t*-value was calculated as

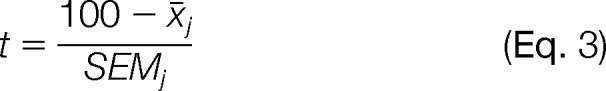

in which *x̄_j_* and *SEM_j_* are, respectively, the mean and standard error of the mean of the relative cell proliferation for peptide aptamer *j*. The hypothesis was accepted for a *p* value (one-tailed) of 0.05 or less.

## RESULTS

### 

#### 

##### Random Mutagenesis

**Fig. 1.**
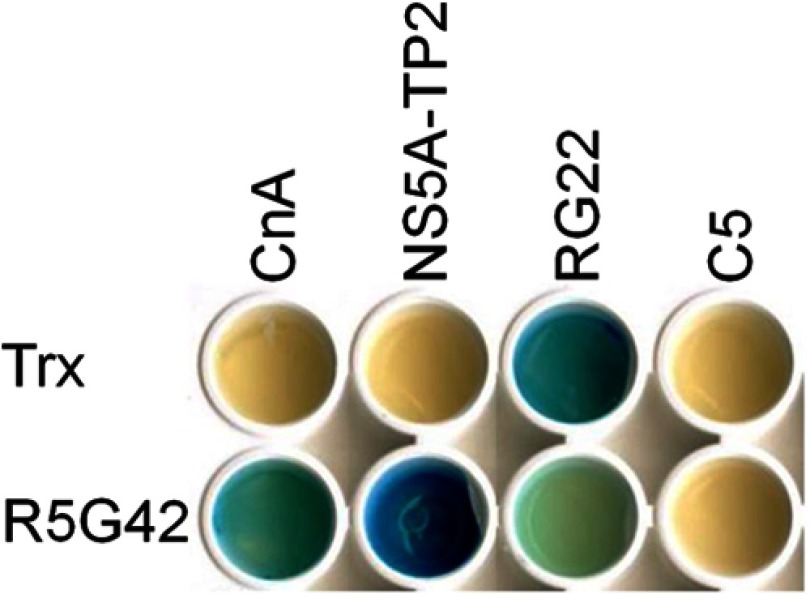
**Specificity of the R5G42 peptide aptamer.** Yeast two-hybrid matrix showing the phenotype of interaction of peptide aptamer R5G42 (11) *versus* the binding region on CnA (amino acids 385–520) and full-length NS5A-TP2. Blue indicates interaction between two proteins; beige indicates the absence of interaction. RG22 is a peptide aptamer that binds to LexA, which is used as an internal positive control for expression of the “bait” plasmid, and C5 is a non-relevant peptide aptamer used as a negative control. Trx is the thioredoxin scaffold alone, which does not interact with the targets (CnA and NS5A-TP2) (see “Experimental Procedures”).

**Fig. 2.**
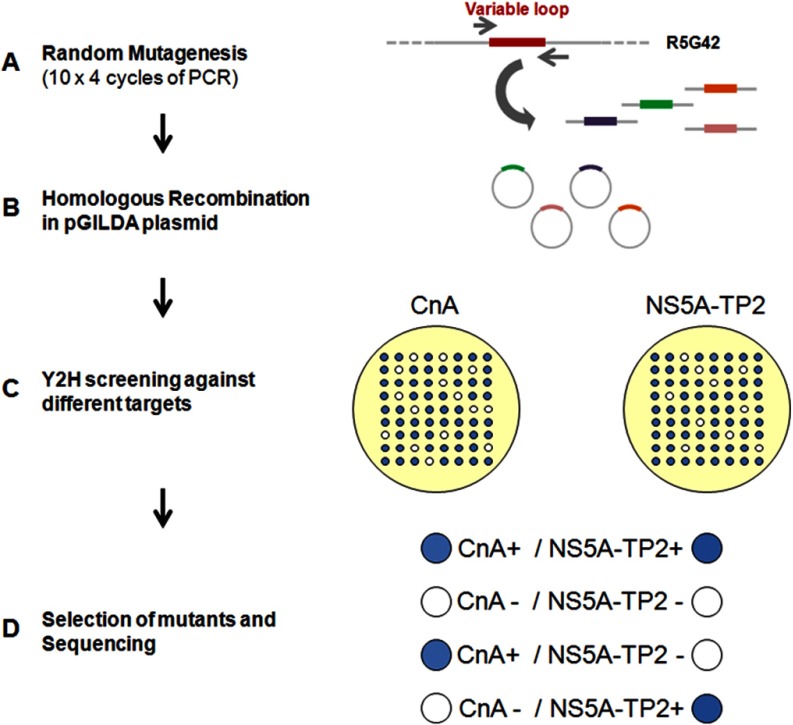
**Workflow for the selection of target-specific peptide aptamers via low frequency random mutagenesis of the variable loop.** *A*, a library of mutant peptide aptamers was generated via low frequency random mutagenesis on the variable loop of the parental peptide aptamer (R5G42). *B*, PCR products were then purified and introduced by means of homologous recombination into the previously linearized pGilda-hTrx plasmid (≈1200 clones). *C*, pGilda-mutant peptide aptamers (225 clones) were used as bait in a Yeast two-hybrid (Y2H) screening *versus* pJG4–5 coding for CnA or NS5A-TP2 (preys) and controls. *D*, 39 mutants with different interaction phenotypes were selected and sequenced for analysis of the mutations.

**Fig. 3.**
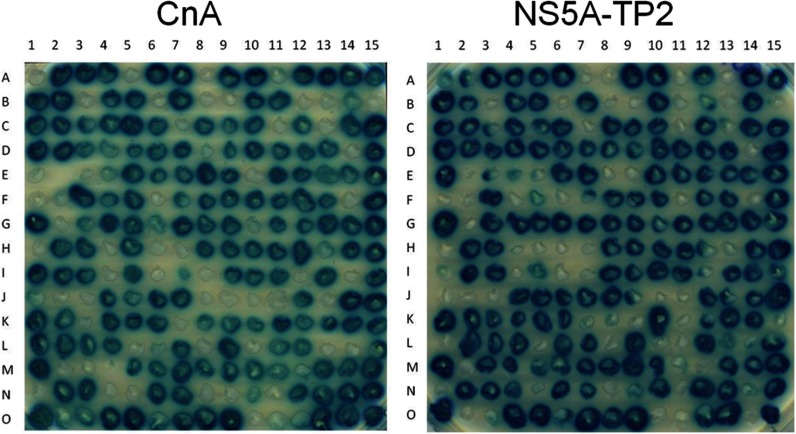
**Example of arrays for analysis of binding specificity of each mutant.** All mutants obtained via random mutagenesis on the variable loop of R5G42 were tested on a 15 × 15 yeast two-hybrid matrix *versus*, respectively, the binding regions on CnA (amino acids 385–520) and full-length NS5A-TP2, as illustrated in Fig. 1, in order to identify peptide aptamers specific for each target. 145 (64%) retained the binding to both targets, 21 (9%) bound to CnA only, 19 (8%) bound to NS5A-TP2, 26 (11%) bound to neither, and 14 (6%) were either not expressed or poorly folded, preventing their detection. Of these mutants, 39 clones were selected according to their phenotypes of interaction in this assay and then sequenced.

**Fig. 4.**
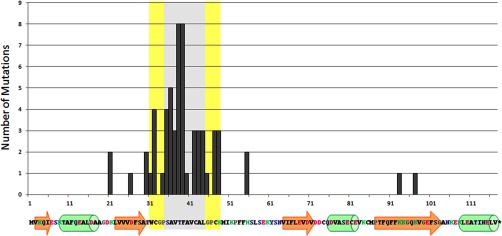
**Overall frequency of mutations observed in the variable loop and scaffold.** Representation of the frequency of mutations as a function of amino acid residue after random mutagenesis of the variable loop, performed as described under “Experimental Procedures.” The sequence of the R5G42 peptide aptamer is indicated under the *x*-axis, with the relative positions of the beta sheets (orange arrows) and alpha helices (green cylinders) derived from the NMR structure of human Trx (PDB-4Trx). The gray zone encompasses the variable loop. The yellow zones indicate the four flanking amino acid residues. 85% of the clones had, as expected, a mutation within the targeted region (variable loop plus the four flanking amino acids).

Peptide aptamer R5G42 recognizes both CnA and NS5A-TP2 (Fig. 1 and Ref. 11). In order to identify peptide aptamers specific for either CnA or NS5A-TP2, a library of mutants was generated by means of random mutagenesis within the variable loop of R5G42 according to the workflow illustrated in Fig. 2. Four amino acids at the N-terminal and four at the C-terminal side of the variable loop were also included in the mutagenized region. Each mutant generated was cloned by means of homologous recombination into the hTrx platform. Approximately 1200 clones were obtained, of which 225 clones were tested in the Y2H mating assay *versus* CnA, NS5A-TP2, and relevant controls in a 15 × 15 matrix (Fig. 3). 145 (64%) retained the binding to both targets; 21 (9%) bound to CnA only, 19 (8%) bound to NS5A-TP2, 26 (11%) bound to neither, and 14 (6%) were either not expressed or poorly folded, preventing their detection. Among these mutants, 39 clones were selected and sequenced according to their interaction phenotype. The frequency of mutation of each residue (Fig. 4) shows that random mutagenesis followed by yeast gap repair created, as expected, mutants primarily within the targeted region (85% of the clones). 30% of the sequenced clones had a single amino acid change specifically in the variable loop. Among the residues of the targeted domain, positions T38 and F39 were the most frequently mutated.

##### Identification of Target-specific Peptide Aptamers

**Fig. 5.**
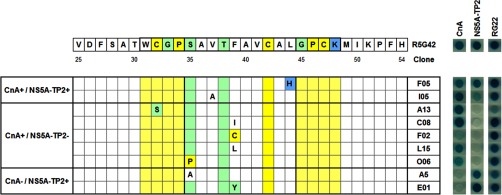
**Position and binding properties of selected single mutations of R5G42.** Single mutations in the region including the variable loop (S35–L44) and four residues N- and C-terminal thereof are represented. Physico-chemical characteristics are color-coded: blue = positive, green = polar, white = apolar, and yellow = special. The bold gray box indicates the variable loop, and the pale yellow region indicates the flanking four residues. The results of a characteristic Y2H mating assay for each clone *versus* CnA, NS5A-TP2, or positive control (RG22) are presented. Blue indicates binding. Translucent indicates no detectable binding. Clones C08 and E01 were used for subsequent tests.

Among the 39 clones that were sequenced, there were 30 unique full-length clones. 16 of these had single amino acid mutations, 10 of which were in the variable loop. Fig. 5 offers a selection of single mutations of the peptide aptamer R5G42 arranged according to binding phenotype, with specific selectivity toward CnA or NS5A-TP2.

Of particular importance, mutants at the amino acid F39 had phenotypes with opposite specificity; that is, different modifications of this residue enabled the selection of peptide aptamers specific for one or the other target. Specifically, the mutation F39**I** generated a CnA-specific peptide aptamer (Apta-C8), whereas the mutation F39**Y** created an NS5A-TP2-specific peptide aptamer (Apta-E1). Thus, amino acid F39 appears to be a key residue implicated in the dual target phenotype. Apta-C8 and Apta-E1 were therefore retained as peptide aptamers specific for CnA and NS5A-TP2, respectively, and were used for further characterization in mammalian cells.

##### Potential Loop Conformations Binding to CnA or NS5A-TP2

Single-point mutations at amino acid positions 35 and 39 within the variable loop of R5G42 are sufficient to change the binding phenotype for the interaction with CnA and NS5A-TP2. Structural biology experiments utilizing NMR or x-ray could provide valuable insights into how mutations at those positions influence the structure of peptide aptamer R5G42 and its complex with the targets CnA and NS5A-TP2. However, data from such experiments are not available yet. We therefore addressed this question with biomolecular computer simulations of R5G42 and four of its mutations (S35P, S35A, F39I, and F39Y).

**Fig. 6.**
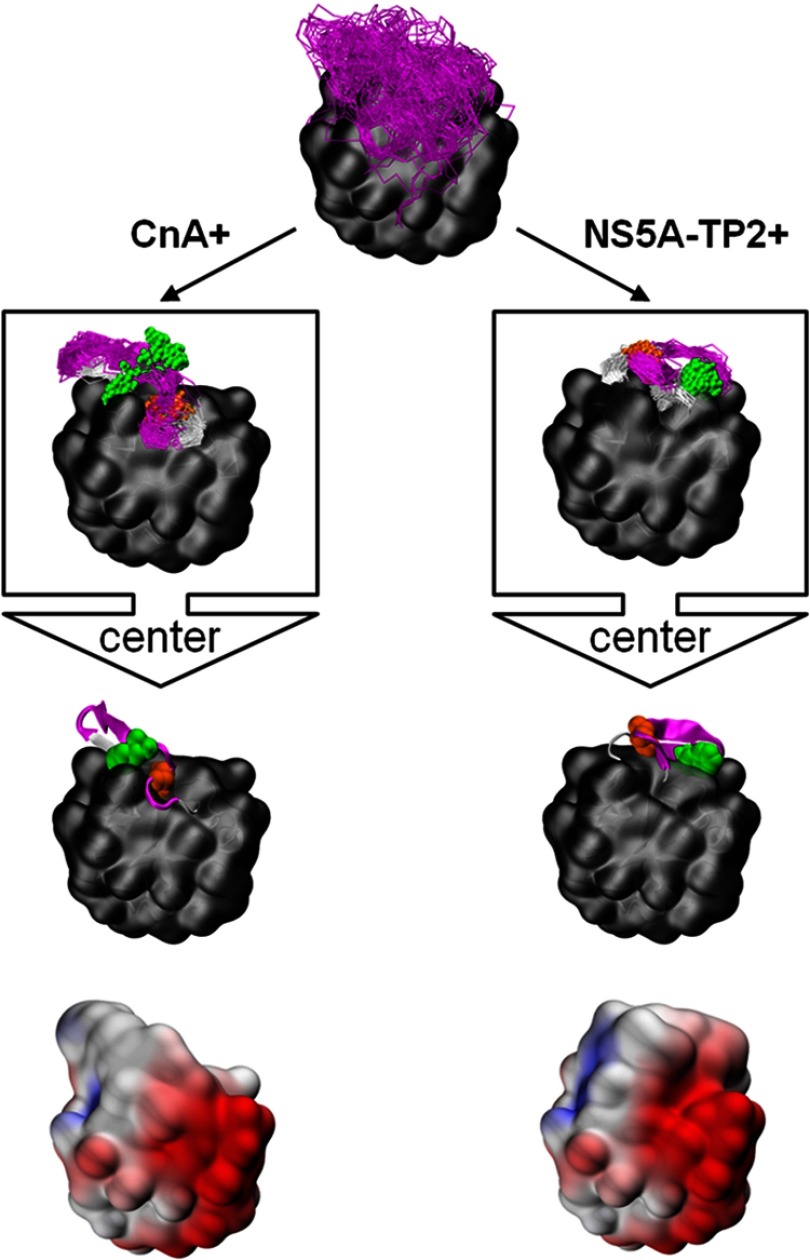
**Loop conformations accessible to peptide aptamer R5G42 and point mutations at residues 35 and 39.** Top: the figure shows an ensemble of 500 randomly picked snapshots from the parallel tempering computer simulation of R5G42 at 310 K. The scaffold is shown as a gray surface, and the backbone of the variable loop (residues 32–47) as magenta trace. Boxes: sub-ensembles for two loop conformers are depicted that are potentially binding CnA (left) and NS5A-TP2 (right). These loop conformers correspond to entries 13 (CnA-binding) and 4 (NS5A-TP2-binding) of Table I. A random selection of about 100 snapshots is shown. The atoms of residues 35 (orange) and 39 (green) are shown as spheres. The center of each cluster is shown together with its electrostatic surface potential as calculated with the PDB2PQR web portal (55) (bottom). For the visualization we used the program VMD (56).

Enhanced sampling of the conformational space accessible to the variable loop, via molecular-dynamics-based parallel tempering with an implicit solvation model, indicated that the peptide aptamer R5G42 exhibits a substantial conformational flexibility, visualized in Fig. 6 (top); more than 19 structurally distinct loop conformers were found within a free-energy span of 2.5 kcal/mol. Mutating residues S35 or F39 changes considerably the population of these conformers (Table I).

For peptide aptamers that bind to the same target and differ by only a single point mutation, it is reasonable to assume that the variable loop adopts a similar conformation when bound to the target. This loop conformation might not be the most prevalent conformation in the unbound state, but it should be accessible without a large penalty (*i.e.* moderate free-energy change) so that its population is sufficient for binding by conformational selection (42, 43) or so that the peptide aptamer can fold as it binds (induced fit) (43, 44). Interestingly, we found only a single loop conformer (entry 13 of Table I) that was populated for all three CnA-binding peptide aptamers. The free-energy difference of conformer 13 with respect to the most populated conformer was significantly smaller for F39I (0.2 kcal/mol) (Apta-C8) than for the other two peptide aptamers, R5G42 and S35P (Apta O06) (2.3 and 1.2 kcal/mol); remarkably, F39I also had a much stronger Y2H binding phenotype than R5G42 and S35P. In conformer 13, the first half of the variable loop interacts with the scaffold as a β-strand; the side chain of residue 35 is buried, and residue 39 is partially solvent exposed and available for interaction (see Fig. 6, left). For the mutations S35A (Apta-A5) and F39Y (Apta-E1), loop conformer 13 is not populated. This could explain their loss of the Y2H binding phenotype for the target CnA.

For the peptide aptamers that bind NS5A-TP2 (*i.e.* R5G42, S35A, and F39Y), there are four populated loop conformers (entries 2, 4, 7, and 9 of Table I). Conformers 2 and 7 are also largely populated in the case of S35P (which does not bind NS5A-TP2). The physical-chemical surface properties of conformer 2 are basically identical regardless of the type of amino acid at position 35 (supplementary Fig. S1), and we would therefore expect the same binding phenotype for R5G42, S35A, and S35P. This is, however, not the case, and conformer 2 can thus be excluded as an NS5A-TP2-binding motif. The same rationale holds for conformer 7 (supplemental Fig. S2). In conformers 4 and 9, the variable loop forms a β-hairpin motif (Fig. 6, right); its orientation is slightly different in the two conformers, but the shape is very similar (and therefore only the more populated conformer 4 is shown in Fig. 6). The β-hairpin-like motif (conformer 4 or 9) is accessible to all three NS5A-TP2-binding peptide aptamers within ≤1.2 kcal/mol of the most populated conformer. The side-chains of residues 35 and 39 interact with the scaffold, but they are also partially solvent exposed. Remarkably, the mutation F39I also populates conformers 4 and 9; nevertheless, F39I does not bind to NS5A-TP2 as observed in the Y2H experiments. Our working hypothesis is that the solvent-exposed face of residue 39 is involved in a π-stacking interaction with the target NS5A-TP2, and its mutation from an apolar, aromatic residue (as with R5G42 and F39Y (Apta-E1), both binding to NS5A-TP2) to an apolar, non-aromatic residue (as with F39I (Apta-C8), F39L (Apta-L15), and F39C (Apta-F02), all not binding to NS5A-TP2) would disfavor the binding.

##### Mapping of Apta-C8 Binding Site on CnA

**Fig. 7.**
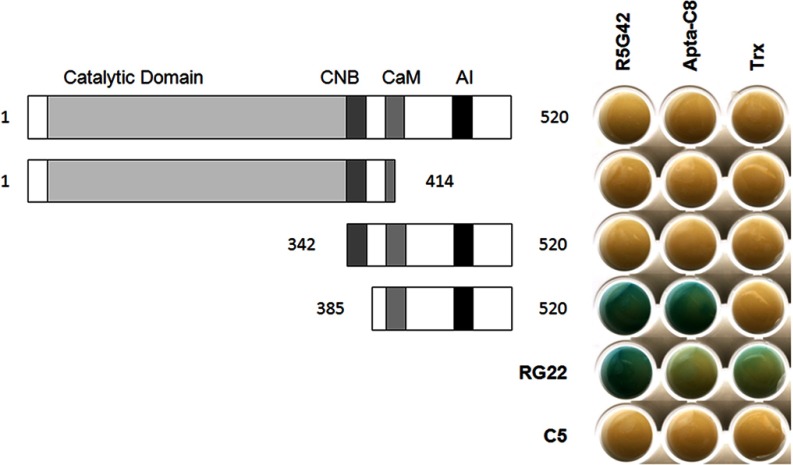
**Identification of the binding domain of Apta-C8 on CnA.** R5G42, Apta-C8 and Trx were evaluated by Y2H for their interaction with different fragments of CnA. The interaction matrix shows that both R5G42 and Apta-C8 bind to the C-terminal region of CnA, in a region that encompasses the Calmodulin binding domain (CaM) and the Auto-Inhibitory domain (AI). The truncation mutants of Calcineurin lacking the Catalytic domain and the Calcineurin B (CNB) binding domain, were constructed by amplification *via* PCR, from a full-length cDNA. Constructs were then cloned into the prey plasmid pJG4-5, by homologous recombination in yeast as described under Experimental Procedures.

An initial step toward elucidating the mechanism of action of Apta-C8 on the activation of calcineurin activity entailed the identification of the specific binding site of the peptide aptamer on the phosphatase. Several deletions of selected portions of the protein were performed. Each construct was designed in order to keep the structure of the different domains of the protein. The truncated forms were then used as preys in a Y2H assay. As shown in Fig. 7, both R5G42 and Apta-C8 showed a binding phenotype with the C-terminal part of calcineurin in a region that includes the CaM binding domain and the AI domain (amino acids 385–520). No interaction was detected when the domains were expressed individually (not shown). Thus, the minimal region for interaction is residues 385–520.

##### Peptide Aptamer–Target Interaction in HeLa Cells

**Fig. 8.**
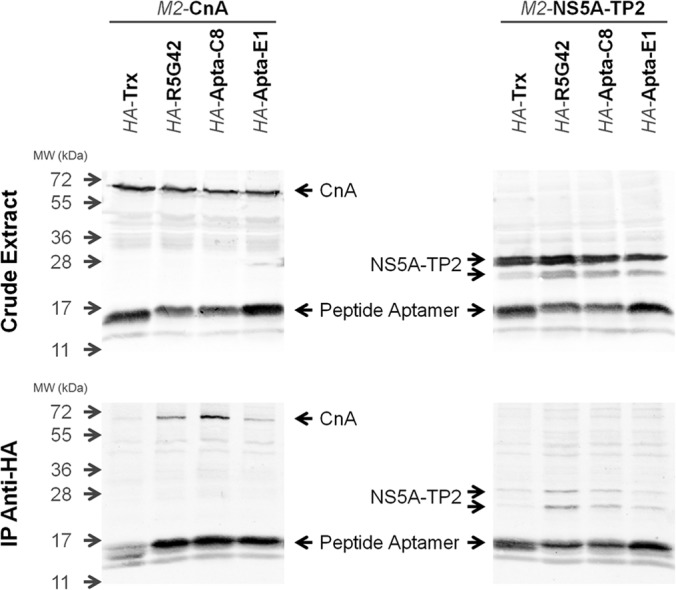
**Validation of interaction in HeLa cells.** Western blot showing the co-immunoprecipitation of CnA or NS5A-TP2 with the various peptide aptamers. Cells were co-transfected with the individual target in the pX2-M2 plasmid coding for CnA or NS5A-TP2 in fusion with the M2 tag and a corresponding expression plasmid, pCI-HA, coding for Trx, R5G42, Apta-C8, or Apta-E1 in fusion with the HA tag. Extracts were prepared and adjusted to 1 mg/ml, immunoprecipitated with anti-HA antibody coupled with magnetic beads, and revealed with anti-HA and anti-M2 antibodies as described under “Experimental Procedures.” The top panel shows the expression level of the recombinant proteins present in 70 μg of extract, and the bottom panel shows the results of the co-immunoprecipitation assays.

To investigate the interaction between the various peptide aptamers and their targets in HeLa cells, we performed an immunoprecipitation followed by analysis via Western blot. Cells were co-transfected with a plasmid carrying an M2-tagged-CnA or NS5A-TP2 and an HA-tagged construct of the various peptide aptamers or Trx control in order to facilitate detection. The immunoprecipitation was performed using an anti-HA antibody coupled to magnetic beads. The presence of the peptide aptamer and the targets in protein extracts and immunoprecipitated samples was determined via Western blotting using mouse monoclonal antibodies toward, respectively, the HA and M2 epitopes, followed by secondary antibody coupled to DyLight 800 (Green Fluorescence) and analysis on an Odyssey imaging system as described under “Experimental Procedures.” As shown in Fig. 8, CnA was co-immunoprecipitated with R5G42 and Apta-C8, whereas the signal observed with Apta-E1 was significantly lower—enough to be indistinguishable from the negative control, Trx. NS5A-TP2 is present in two forms. The higher molecular weight band is the full-length protein and is most abundant in the crude extract. The lower molecular weight form, ∼3 kDa less than the upper band, is much less abundant. Both forms appear to have been co-immunoprecipitated with R5G42, Apta-C8, and Apta-E1. Both bands were weaker in the Apta-C8 and Apta-E1 lanes than in the R5G42 lane, but stronger than what was observed with the Trx control, and even more so relative to the crude extract. It appears that the lower molecular weight band was enriched in the samples brought down with the peptide aptamers relative to the Trx control.

##### Apta-C8 Stimulates Nuclear Translocation of NFAT in HeLa Cells

**Fig. 9.**
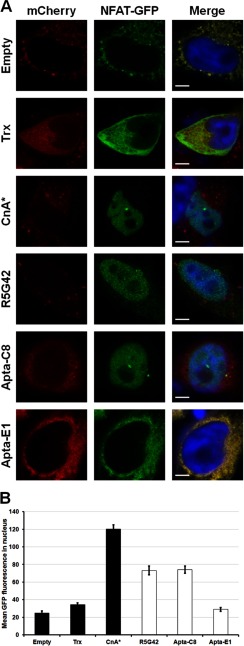
**Nuclear translocation of NFATc1.** *A*, the presence of green fluorescence in the nuclei of transfected cells indicates that R5G42 and Apta-C8 induce the nuclear translocation of NFATc1-GFP, an indication of the activation of CnA and the dephosphorylation of NFAT. Constitutively activated CnA (CnA*) was used as a positive control for the NFAT-translocation assay. The pictures are a compilation from images generated during a characteristic experiment. mCherry: indirect reporter for all of the recombinant protein targets and controls (Trx, R5G42, Apta-C8, Apta-E1, CnA*) as expressed under the control of an internal ribosome entry site on the same mRNA as the proteins of interest. *B,* quantitation of NFAT-GFP in the nucleus performed as described under “Experimental Procedures” (mean ± S.E.; data compiled from two independent experiments: empty vector *n* = 16; Trx *n* = 16; R5G42 *n* = 20; Apta-C8 *n* = 22; Apta-E1 *n* = 18; CnA* *n* = 34).

In order to validate whether any of the peptide aptamers were able to activate endogenous CnA, as the parental peptide aptamer R5G42 is, we evaluated their capacity to provoke the nuclear translocation of NFATc1 in HeLa cells. As shown in Fig. 9*A*, the expression of a constitutively active form of calcineurin (CnA*) induces a translocation of NFATc1 into the nucleus, whereas with the negative controls, Empty vector and Trx, NFATc1 is localized exclusively in the cytoplasm. In cells transfected with R5G42 or Apta-C8, the NFAT-GFP is visible in the nucleus, confirming the activation of NFAT, even if it is weaker than that of CnA*. Note that with Apta-E1, the peptide aptamer specific for NS5A-TP2, no nuclear translocation of NFAT is observed. Results obtained from the analysis of multiple (16 to 34) cells under each condition are illustrated in the bar graph presented in Fig. 9*B*.

##### Characterization of the Anti-proliferative Properties of the Target-specific Mutants

**Fig. 10.**
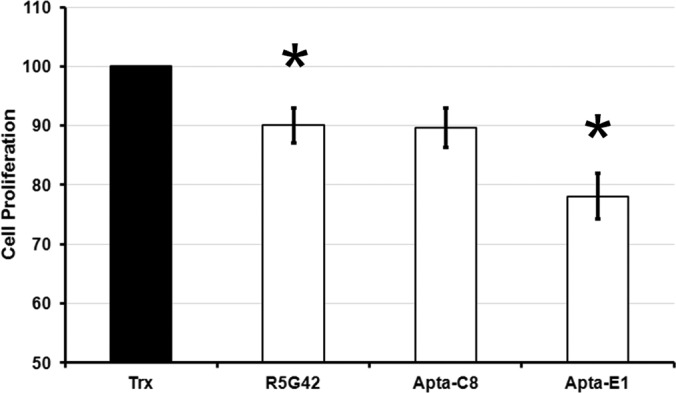
**Characterization of the peptide aptamer anti-proliferative activity.** XC cells were labeled with CMTMR and then transfected with pCI-HA plasmids coding for Trx, R5G42, Apta-C8, or Apta-E1 peptide aptamers. Analyses were performed on living cells expressing the various peptide aptamers at 48 h post-transfection and compared with the analyses at *t* = 0 h. Anti-proliferative activity was determined via measurement of the geometric mean of the CMTMR labeling in transfected cells (revealed by peptide aptamer/Trx expression). For each condition, cell proliferation was calculated using the following formula: 100 × (signal *t* = 0 h − signal *t* = 48 h) of peptide aptamer/(signal *t* = 0 h − signal *t* = 48 h) of Trx. Thus, the maximum proliferation (arbitrarily set at 100) corresponds to the maximum loss of CMTMR signal between *t* = 0 h and *t* = 48 h for Trx expressing cells. The three peptide aptamers studied provoke a significant decrease in cell proliferation relative to cells expressing Trx. The results presented correspond to the average of three to four independent experiments (Trx, R5G42, and Apta-E1 *n* = 4, Apta-C8 *n* = 3). Error bars represent the standard error of the mean; * indicates *p* < 0.05. The calculated *p* values for the statistical significance of the observed shifts relative to Trx (see “Statistical Analyses”) are 0.03 (R5G42), 0.06 (Apta-C8), and 0.01 (Apta-E1).

The target-specific mutants obtained were used to investigate the relative contributions of CnA and NS5A-TP2 to the observed anti-proliferative activity of R5G42. Thus we challenged R5G42, C8, A5, and E1 in an anti-proliferative assay (11). XC cells were labeled with CMTMR and then transfected with pCI-HA plasmids coding for the various peptide aptamers or the inactivated scaffold Trx, which was the negative control. Anti-proliferative activity was then determined as a reduction of cell proliferation relative to the Trx-expressing cells over the 48-h time span of the experiment as described in “Experimental Procedures.” The results of this approach are presented in Fig. 10. Clearly, all challenged peptide aptamers were able to slow cell proliferation relative to the empty Trx scaffold, with Apta-E1 being the most efficient. The *p* values (for test statistics with a *t*-distribution, see “Statistical Analyses”) ranged between 0.01 and 0.06 and thus were below (or close to) the critical value of 0.05. Thus, both CnA and NS5A-TP2 appear to be involved in the R5G42-specific reduction in cell proliferation.

## DISCUSSION

With the development of systems biology approaches and the implementation of global analysis techniques for understanding physiological processes, it is essential to have a means of identifying key pathways and proteins therein whose activity is crucial for a given biological response (45). Additionally, precision tools with which to probe pathways and modulate protein activity enable focused investigation into the mechanisms by which the processes are regulated. The use of siRNA to screen for proteins that modulate biological responses has offered a wealth of information in this regard, yet it remains a first level of appreciation of such parameters. Indeed, a reduction in the expression of a protein or its complete removal (knock-out of the encoding gene) reduces or eliminates the interaction with all of its partners. It would ideally be necessary to be able to perturb interactions selectively in order to be able to achieve the next level of precision in analyzing protein networks.

The implementation of peptide aptamer technology offers this opportunity. Peptide aptamers are small combinatorial proteins that can be selected for their binding to a specific target and then evaluated for their potential to modulate the protein function, including the identification of binding sites. This approach allows the determination of the role of a known protein in a pathway or biological response (7, 46). Alternatively, it is possible to directly identify peptide aptamers that modulate a particular pathway or physiological response, and subsequently their targets, using a functional selection approach (11). This can offer interesting insights that would not necessarily come from the bioinformatic analysis of known pathways.

As an example, in an anti-proliferative functional selection, we previously identified peptide aptamer R5G42 (11). Interestingly, R5G42 has dual target specificity. It binds to CnA, a serine/threonine protein phosphatase (17), and to NS5A-TP2, a protein trans-activated by hepatitis C virus NS5A (12). The objective of the present study was to identify peptide aptamers specific for either CnA or NS5A-TP2 in order to be able to further dissect their roles in signaling pathways. This was achieved through the implementation of low frequency random mutagenesis directed toward the variable loop of the original peptide aptamer R5G42, followed by Y2H evaluation of the specificity of the selected clones for the different targets.

### 

#### 

##### Low Frequency Random Mutagenesis Allowed Dissection of the Target Specificity of R5G42

The low frequency random mutagenesis approach described herein generated a library of 1200 mutants, from which 225 were randomly evaluated for their capacity to bind one or the other of the targets. Of the 39 clones that were subsequently sequenced, we focused on those showing a single mutation in the variable loop. The analysis of the frequency of mutation for each residue showed that amino acid positions T38 and F39 were the most frequently mutated. In particular, mutations in the codon for amino acid F39 offered strategic insight important for the selectivity of the binding with one or the other target. Notably, for example, the mutation F39I confers the specificity for CnA (Apta-C8), whereas the mutation F39Y confers the specificity for NS5A-TP2 and a subsequent loss of binding with CnA (Apta-E1).

##### Insight into the Binding Specificity Offered by Molecular Modeling Combined with Y2H

For the binding of the peptide aptamer to NS5A-TP2, the aromaticity of the amino acid at position 39 is decisive. Mutating the aromatic, apolar F39 to a non-aromatic, apolar amino acid leads to the loss of the NS5A-TP2 binding phenotype. In contrast, when F39 is mutated to tyrosine, an aromatic, less apolar, larger amino acid, the binding phenotype is maintained. This points to π-stacking effects involved in either the direct binding interaction of F39 with NS5A-TP2 or the stabilization of an NS5A-TP2-specific conformation of peptide aptamer R5G42. In the conformation potentially binding NS5A-TP2 (as obtained by integrating the results from Y2H into the analysis of the computer simulations; see Fig. 6, right), amino acid residue 39 interacts with the scaffold and the rest of the loop, but there is no π-stacking involved. The other face of the side-chain of residue 39 is available for interaction with the target. Thus, the former case (direct interaction of residue 39 with the target) seems more likely.

Regarding the binding of the peptide aptamer to CnA, the apolarity of the amino acid at position 39 is crucial. Maintaining hydrophobicity ensures binding to CnA (F39I, F39L, F39C), whereas the addition of a polar hydroxy-group (F39Y) causes a loss of the binding phenotype. The conformation potentially binding CnA (Fig. 6, left) displays a partially solvent-exposed residue 39. When the amino acid at position 39 is binding to an aliphatic pocket of CnA, its mutation to a less apolar and larger residue (F39Y) could lead to a decrease in the binding affinity.

We have demonstrated through mapping studies (see above) that the AI domain and the CaM binding domain both are needed to achieve the binding of Apta-C8 to CnA. This is in agreement with the previously reported binding of R5G42 (11). This could mean that either both domains are involved in the interaction with the peptide aptamer, or the presence of both domains is necessary in order for the region to adopt a conformation recognized by the peptide aptamer (intramolecular allosteric regulation) (47).

In principle, the peptide aptamers can be present in two different states, oxidized (with a di-sulfur bridge between C32 and C47) or reduced (with two thiol groups). Construct A13 in Fig. 5 (mutation C32S, in which no di-sulfur bridge is possible because one of the cysteines has been mutated) indicates that the oxidized state is not required for the binding of this peptide aptamer to CnA. For the binding to NS5A-TP2, however, it is possible that the oxidized state is required.

Overall, this analysis offers insight into the binding of each target and sets the stage for future investigations into the structural basis for the interactions.

##### Both Target-specific Peptide Aptamers Inhibit Cell Proliferation

The characterization of the effect of our peptide aptamers on cell proliferation showed that both targets, CnA and NS5ATP2, are implicated in the reduction of cell proliferation observed with R5G42 in XC cells. This result indicates that R5G42 is able to interact with two different cellular proteins (45) that contribute to this cell proliferation phenotype. The generation of peptide aptamers specific for one or the other target has allowed an appreciation of the contribution of binding to NS5A-TP2/HDDC2. Our work provides the first indication of a potential role for NS5A-TP2/HDDC2 in the regulation of cell proliferation. We further provide the means with which to investigate this cellular response via the use of Apta-E1.

NS5A-TP2 is a protein whose function has not been characterized. The presence in its sequence of an HD domain suggests its potential involvement in nucleic acid metabolism and signal transduction (13). However, as of this writing, other than our observations reporting the reduction of cell proliferation once bound with a dual target or single target specific peptide aptamer, nothing is known about its biological function. The identification of a peptide aptamer that binds NS5A-TP2, modulating its activity, therefore represents a crucial tool for dissecting its role in cellular signaling pathways. Further investigations of the effect of Apta-E1 on cellular responses can thus offer important information that could be used to determine the functional role of NS5A-TP2.

In contrast, calcineurin is a well-characterized protein phosphatase (18). CnA is ubiquitously expressed and is involved in the regulation of different biological processes, such as the development and regulation of skeletal (20) and cardiac muscle (21), the nervous system (19), and the immune system. Upon calcium activation, CnA modulates the expression of specific genes via the dephosphorylation, and thus activation, of transcription factors, the most studied of which is NFAT. Although inhibitors of this phosphatase are already well known, such as cyclosporin A and FK506 (Tacrolimus), to date no molecule able to directly bind and *activate* calcineurin has been identified other than R5G42, which has dual target specificity (see, however, Ref. 25).

Our peptide aptamer Apta-C8 is the first exogenous molecule reported that specifically binds CnA, up-regulating its activity. It represents, thus, an important tool for studying the impact of the *activation* of endogenous CnA in the regulation of different signaling pathways in, for example, the immune, skeleto-muscle, and nervous systems. The modulation of CnA activity has attracted much attention because of the multiple facets of its action. Until now, most studies have depended on the use of small molecule inhibitors of CnA, such as cyclosporin A and FK506, or the overexpression of constitutively activated CnA (CnA*) to determine causal relationships between physiological responses and CnA activation. The use of the peptide aptamer Apta-C8 is expected to enable, in several experimental situations, the study of the effect of the heretofore unattainable direct activation of CnA in model systems.

Given the crucial role of calcineurin in different signaling pathways and, moreover, considering the effect of the modulation of its activity in numerous pathologies, such as muscle atrophy (48, 49) and dystrophy (26) and neurodegenerative diseases (28), we focused principally on the further characterization of Apta-C8.

##### Co-immunoprecipitation of Peptide Aptamer–Target Pairs in Mammalian Cells

The interaction of both R5G42 and Apta-C8 with CnA observed in Y2H studies was confirmed via co-immunoprecipitation in HeLa cells. Apta-E1, however, though well expressed, appeared to behave similarly to the Trx control, again in agreement with the Y2H results. Regarding NS5A-TP2, R5G42 appeared to have the most robust association overall, and Apta-C8 was much weaker, followed by Apta-E1 and Trx. Interestingly, the lower band appeared to be more strongly associated with the peptide aptamers than the signal observed with the Trx control. This is in direct contrast to the relative expression levels in the crude extract. At present we do not know whether this is a different splice form or a truncated version of NS5A-TP2. Further investigation will be necessary in order for this association to be understood. Nevertheless, the phenotype observed using the Y2H analysis remains valid in this regard.

##### Activation CnA by Peptide Aptamers Is Visualized by Means of Nuclear Translocation of NFAT

One of the key hallmarks of the activation of CnA is the nuclear translocation of NFAT. This is due to the dephosphorylation of selected residues, which results in the hiding of a nuclear export sequence, allowing the protein to accumulate in the nucleus. Peptide aptamer R5G42 was shown to be able to activate CnA *in vitro* and in cultured cells (11, 25). Here, we observed that R5G42 co-expression with NFAT1c-GFP resulted in nuclear translocation of the latter, mimicking the effect of constitutively activated CnA (CnA*), albeit at a lesser overall amplitude. Similar to R5G42, Apta-C8 was able to stimulate the nuclear translocation of NFATc1, indicative of the direct activation of CnA and subsequent dephosphorylation of NFAT. In contrast, neither Trx nor Apta-E1 co-expression was able to stimulate this response, similar to the negative control (Empty vector), thus offering functional corroboration of the Y2H binding phenotype for all of the peptide aptamers and Trx.

##### Mapping of the Peptide Aptamer Binding Region

The peptide aptamer binding region on CnA was identified by observing the binding phenotype of truncations of the protein in Y2H, as described elsewhere (11, 25). We have established that the binding region of Apta-C8 on the CnA sequence is restricted to the C-terminal part, in a region that encompasses the CaM binding domain and the AI domain, as was observed for R5G42.

This peptide aptamer–minimal binding domain pair can be used for the identification of small molecules that displace the peptide aptamer from the target (9). Such molecules could then be evaluated for their capacity to exert the same activating function as Apta-C8 on CnA. This could lead to the development of a novel therapeutic approach to alleviate the pathological effects of selected diseases such as Duchenne muscular dystrophy (50, 51), amyotrophic lateral sclerosis (52), and Alzheimer's disease (27, 28).

##### Postulated Mechanism of Calcineurin Activation by R5G42 and C8

The C-terminal AI domain of CnA regulates the phosphatase activity of calcineurin. Under basal calcium conditions, the AI domain occupies the catalytic site and thereby blocks substrate binding and prevents dephosphorylation (53). With increased calcium concentrations during signaling, the binding of calcium and calmodulin to calcineurin displaces the AI domain from the catalytic site and stimulates calcineurin's phosphatase activity (54). Our Y2H experiments with truncated forms of CnA have demonstrated that both the AI domain and the calmodulin binding domain are essential for the binding of R5G42 and Apta-C8. We postulate, therefore, that the binding of peptide aptamers R5G42 and Apta-C8 to CnA leads to a reduction of the intramolecular binding of the AI domain to the catalytic site, via direct interaction with the AI and/or interaction with a more distant site that induces a conformational change that displaces the AI domain. This increases the fraction of enzymes with an unoccupied catalytic site available for substrate dephosphorylation, mimicking the activation effect of calcium/calmodulin binding to calcineurin upon signaling.

In summary, the implementation of peptide aptamer technology described herein has allowed the development of two precision tools for investigating the role of their respective targets. Apta-C8 will enable investigation of the effect and, eventually, therapeutic potential of the direct activation of endogenous CnA, a well-known target characterized therapeutically for its inhibition. Apta-E1 will be a unique resource for understanding the role and mechanism of action of an essentially unknown target, NS5A-TP2, in cellular processes and pathological situations. These studies underline the pertinence of functional selection using peptide aptamers.

## Supplementary Material

Supplemental Data

## Footnotes

^1^ The abbreviations used are:

AI: auto-inhibitory
Apta: peptide aptamer
CaM: calmodulin
CnA: calcineurin A
HDDC2: HD domain containing 2
NFAT: nuclear factor of activated T cells
NS5A-TP2: hepatitis C virus non-structural protein 5A-transactivated protein 2
Y2H: yeast two-hybrid.
