# Supplementary material for: Calcineurin A versus NS5A-TP2/HD Domain Containing 2: A Case Study of Site-directed Low-frequency Random Mutagenesis for Dissecting Target Specificity of Peptide Aptamers
Source: Mol Cell Proteomics. 2013 Apr 10;12(7):1939–52. doi: 10.1074/mcp.M112.024612 (PMC3708177; doi:10.1074/mcp.M112.024612)
Supplement: Supplemental Data [file supp_12_7_1939__index.html]

Calcineurin A vs NS5A-TP2/HDDC2: a case study of site-directed low-frequency random mutagenesis for dissecting target specificity of peptide aptamers — Calcineurin A versus NS5A-TP2/HD Domain Containing 2: A Case Study of Site-directed Low-frequency Random Mutagenesis for Dissecting Target Specificity of Peptide Aptamers — Dissecting Target Specificity of Peptide Aptamers — Supplemental Data 

# Calcineurin A *versus* NS5A-TP2/HD Domain Containing 2: A Case Study of Site-directed Low-frequency Random Mutagenesis for Dissecting Target Specificity of Peptide Aptamers

## Supplemental Data

**Files in this Data Supplement:**

- Supplemental Figure S1 - The electrostatic surface potential for conformer 2 of peptide aptamer R5G42, R5G42 with mutation S35A (Apta-O06), and R5G42 with mutation S35P (Apta-A05)
- Supplemental Figure S2 - The electrostatic surface potential for conformer 7 of peptide aptamers R5G42, R5G42 with mutation S35A (Apta-O06), and R5G42 with mutation S35P (Apta-A05)
