# Supplementary material for: Calcineurin A versus NS5A-TP2/HD Domain Containing 2: A Case Study of Site-directed Low-frequency Random Mutagenesis for Dissecting Target Specificity of Peptide Aptamers
Source: Mol Cell Proteomics. 2013 Apr 10;12(7):1939–52. doi: 10.1074/mcp.M112.024612 (PMC3708177; doi:10.1074/mcp.M112.024612)
Supplement: Supplemental Data [file supp_M112.024612_mcp.M112.024612-2.pdf]

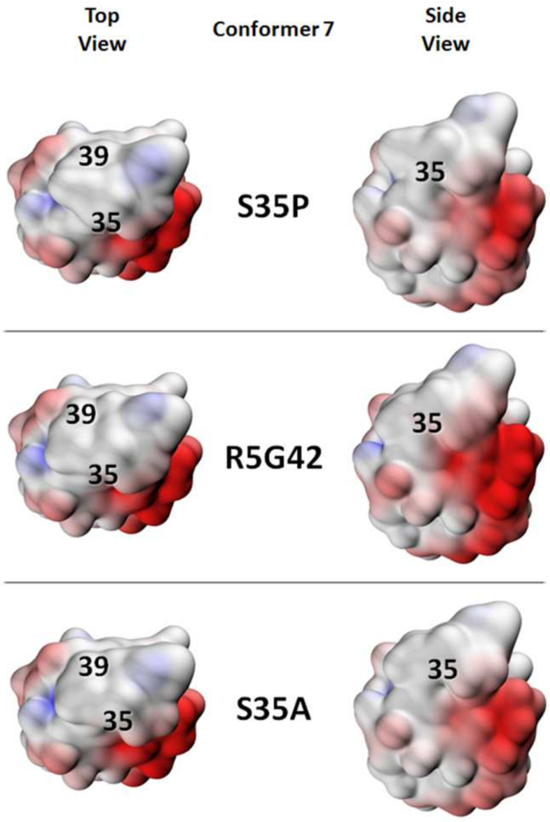

Figure S2. The electrostatic surface potential for conformer 7 of peptide aptamers R5G42, R5G42 with mutation S35A (Apta-O06), and R5G42 with mutation S35P (Apta-A05). The potential ranges from -5 (red) to +5 (blue) kT/e and was calculated with the PDB2PQR web portal (*cf.* main manuscript for references). Note, the yeast two-hybrid experiments indicate that the loop with the mutation S35P does not bind to NS5A-TP2, whereas R5G42 and the variable loop with mutation S35A both bind to NS5A-TP2. Since the electrostatic surface properties of conformer 7 appear to be essentially identical for all three peptide aptamers, and its population is also similar for all three cases, this loop conformer has been ruled out as being a NS5A-TP2-binding motif since it would yield a matching NS5A-TP2-binding phenotype for R5G42, R5G42 with the point mutation S35P, and R5G42 with the point mutation S35A, again, which was not observed experimentally.
